# The Expression Profile of Dental Pulp-Derived Stromal Cells Supports Their Limited Capacity to Differentiate into Adipogenic Cells

**DOI:** 10.3390/ijms21082753

**Published:** 2020-04-15

**Authors:** Letícia Fracaro, Alexandra C. Senegaglia, Roberto H. Herai, Amanda Leitolis, Lidiane M. Boldrini-Leite, Carmen L. K. Rebelatto, Paul J. Travers, Paulo R. S. Brofman, Alejandro Correa

**Affiliations:** 1 Core for Cell Technology, School of Medicine, Pontifícia Universidade Católica do Paraná—PUCPR, Curitiba, Parana 80215-901, Brazil; leticiafracaro@gmail.com (L.F.); alexandra.senegaglia@pucpr.br (A.C.S.); lidiane.leite@pucpr.br (L.M.B.-L.); carmen.rebelatto@pucpr.br (C.L.K.R.); 2 Graduate Program in Health Sciences (PPGCS), School of Medicine, Pontifícia Universidade Católica do Paraná—PUCPR, Curitiba, Parana 80215-901, Brazil; roberto.herai@pucpr.br; 3 Laboratory of Basic Biology of Stem Cells, Carlos Chagas Institute, Fiocruz-Parana, Curitiba, Parana 81350-010, Brazil; aleitolis@gmail.com; 4 Centre for Regenerative Medicine, University of Edinburgh, Edinburgh EH16 4UU, Scotland, UK; Paul.Travers@ed.ac.uk

**Keywords:** ADSCs, DPSCs, adipogenesis, transcription profile, WNT/BMP pathways

## Abstract

Mesenchymal stromal cells (MSCs) can self-renew, differentiate into specialised cells and have different embryonic origins—ectodermal for dental pulp-derived MSCs (DPSCs) and mesodermal for adipose tissue-derived MSCs (ADSCs). Data on DPSCs adipogenic differentiation potential and timing vary, and the lack of molecular and genetic information prompted us to gain a better understanding of DPSCs adipogenic differentiation potential and gene expression profile. While DPSCs differentiated readily along osteogenic and chondrogenic pathways, after 21 days in two different types of adipogenic induction media, DPSCs cultures did not contain lipid vacuoles and had low expression levels of the adipogenic genes proliferator-activated receptor gamma (PPARG), lipoprotein lipase (LPL) and CCAAT/enhancer-binding protein alpha (CEBPA). To better understand this limitation in adipogenesis, transcriptome analysis in undifferentiated DPSCs was carried out, with the ADSC transcriptome used as a positive control. In total, 14,871 transcripts were common to DPSCs and ADSCs, some were unique (DPSCs: 471, ADSCs: 1032), and 510 were differentially expressed genes. Detailed analyses of overrepresented transcripts showed that DPSCs express genes that inhibit adipogenic differentiation, revealing the possible mechanism for their limited adipogenesis.

## 1. Introduction

Mesenchymal stromal cells (MSCs) are multipotent adult cells and are functionally defined as having the following features: capacity for self-renewal, the ability to differentiate into adipogenic, osteogenic and chondrogenic cell lines [1] and extensive paracrine and immunomodulatory activities [2]. MSCs can be isolated from several tissues, including bone marrow, umbilical cord and dental tissues [3]. However, depending on the source, MSCs may have different embryonic origins; for example, adipose tissue-derived stromal cells (ADSCs) have a mesodermal origin, and dental pulp-derived stromal cells (DPSCs) may have an ectodermal origin [4]. Adipogenesis occurs throughout life—in the embryo, the adipocyte lineage originates from mesenchymal progenitors that form adipocyte or pre-adipocyte precursor cells that have not yet accumulated lipids and later differentiate into mature adipocytes containing lipid vacuoles [5]. Published data are conflicting about the potential and the time required for DPSCs to achieve in vitro adipogenic differentiation. Some studies have reported that DPSCs can differentiate into adipocytes as efficiently as other MSCs. However, these studies neither clearly showed the presence of cells with lipid vacuoles after differentiation nor provided cell differentiation efficiency/quantification, and their conclusions were based solely on microscopic identification of a few differentiated cells [6,7,8].

A valuable, yet under-utilised, complementary method of assessing adipogenic differentiation involves evaluating the expression of specific molecular markers of adipocytes, such as peroxisome proliferator-activated receptor gamma (PPARG) and lipoprotein lipase (LPL) [8,9,10]. During the initiation of differentiation and maturation of adipocytes, a transcriptional cascade occurs with activation of multiple factors that can alter the expression of the receptor activated by PPARG proliferators. The final phase of differentiation is characterised by the appearance of lipid droplets and the expression of lipid storage proteins such as: fatty acid-binding protein 4 (FABP4) and LPL [11].

Signalling pathways such as the bone morphogenetic protein (BMP) and WNT pathways have been shown to be involved in the commitment or inhibition of MSCs to the adipocyte lineage [12]. The WNT signalling pathway regulates MSC maintenance, proliferation, fate determination and preadipocyte differentiation [13]. Additionally, it has been demonstrated that both canonical and noncanonical BMP signalling pathways are important in determining MSC differentiation [14]. It is thus essential to determine the expression of the components of these pathways in undifferentiated DPSCs.

In this study, we characterised the transcriptome of DPSCs in comparison with a well-defined mesodermal-origin MSC with strong adipogenic capacity as a control to understand whether the embryonic origin determines a baseline expression profile that could correlate with the differentiation potential. Although our results suggest that DPSCs poorly meet the defined criteria for bona fide multipotent MSCs, DPSCs are nonetheless an interesting cell type with a putative neural potential worth further study.

## 2. Results and Discussion

Inconsistent evidence about the adipogenic capacity of DPSCs demands a more thorough molecular characterisation of these cells for comparison with MSCs, such as ADSCs, which are undoubtedly able to differentiate into adipocytes. To this end, we performed four levels of comparison: microscopic characterisation of ADSCs and DPSCs to evaluate morphology and adhesion to plastic, immunophenotypic analysis following the recommendation of the Mesenchymal and Tissue Stem Cell Committee of the International Society for Cellular Therapy (ISCT) for multipotent MSCs [1], evaluation of adipocytic, chondrocytic and osteoblastic differentiation capacities of both cell types and high-throughput sequencing for transcriptomic analysis.

### 2.1. DPSCs Are Immunophenotypically and Morphologically Similar to ADSCs and at Least Bipotent

**Figure 1:**
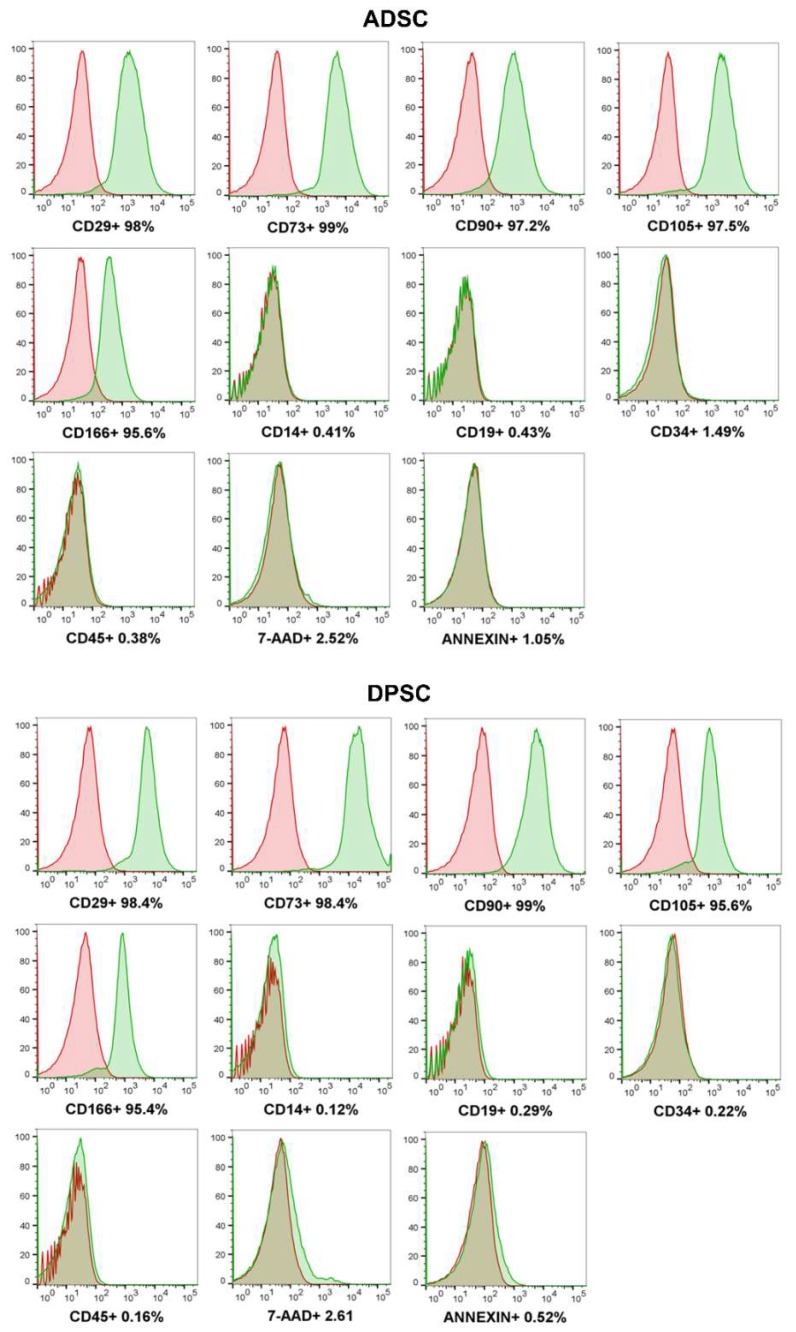
Mesenchymal stromal cell (MSCs) characterisation. Immunophenotypic analysis by flow cytometry of representative ADSCs and DPSCs samples. Green histograms indicate the percentage of the population positive for each antibody, while red histograms indicate the isotype control of the antibodies. ADSCs: adipose tissue-derived stromal cells, DPSCs: dental pulp-derived stromal cells.

Immunophenotypic characterisation of cell surface antigens markers on the ADSCs (*n* = 3) and DPSCs (*n* = 3) samples (Figure 1) revealed that more than 95% of the cells were positive for CD29, CD73, CD90 and CD105, and showed negative or reduced (<5 %) expression for CD14, CD19, CD34 and CD45 [15,16,17]. The results for 7-AAD and Annexin V demonstrated that the cells were viable and exhibited low levels of apoptosis/necrosis. The expression of CD166, an antigen that is not required by the ISCT, yet is considered an MSC marker, was found in >95% of the cells from both sources. Accordingly, other studies have observed positive expression of CD166 in DPSCs [18] and ADSCs [19,20].

**Figure 2:**
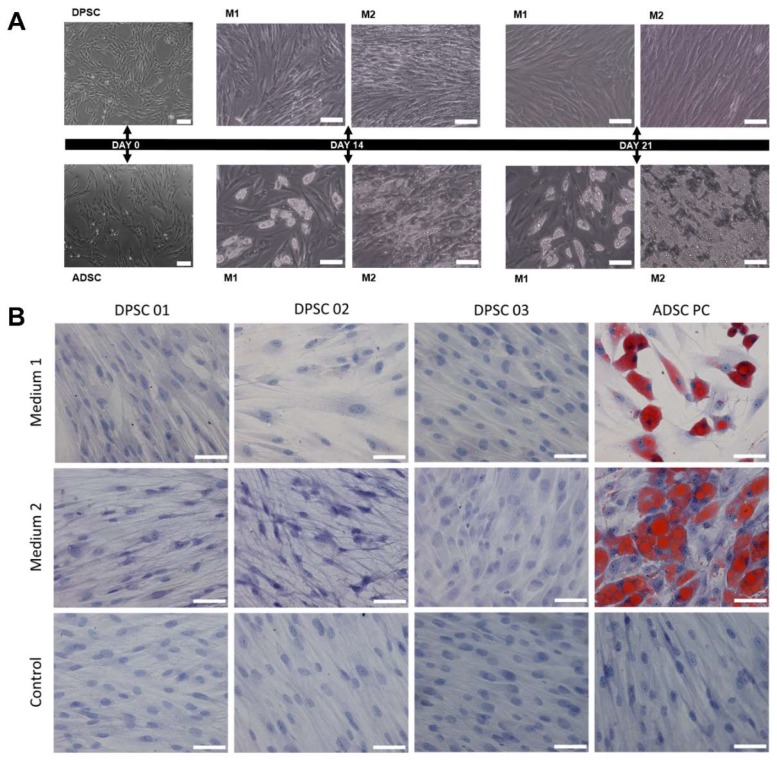
Adipogenic differentiation of MSCs. (**A**) Morphological analysis of the cells on days 0, 14 and 21 after induction for adipogenic differentiation in a representative sample. On days 14 and 21, the presence of lipid vacuoles is observed only in the ADSCs (positive control). Scale bar: Day 0: 20 µm, Days 14 and 21: 100 µm. (**B**) In vitro adipogenic differentiation: comparison between the positive control (PC) (ADSCs) and three samples of DPSCs. Staining: Oil Red O. Scale bar: 50 µm. MSCs: mesenchymal stromal cells; ADSCs: adipose tissue-derived stromal cells, DPSCs: dental pulp-derived stromal cells. M1: medium 1, commercial culture medium, M2: medium 2, custom culture medium.

Visual observation under brightfield microscopy showed that both cell types have fibroblastic morphology and a capacity to adhere to plastic, with no observable differences between the two cell types (Figure 2A).

### 2.2. DPSCs do not Differentiate into Adipocytes After 21 Days of Induction Using Two Different Protocols

Evaluation of the differentiation into the three lineages considered by the ISCT as integral to the definition of MSC showed that both DPSCs and ADSCs differentiated into osteoblasts, as indicated by the presence of calcium crystals after 21 days of induction, and differentiated into chondrocytes, as indicated by the observation of cuboidal cells and gaps around the young chondrocytes and intracellular matrix mucopolysaccharides. In the negative control samples, which were cultured without the induction media, none of these characteristics were observed (Figure 2B and Supplementary Figure S1). The same results have already been obtained in other studies [8,21]. With respect to adipocyte differentiation, however, differences between DPSCs and ADSCs became apparent (Supplementary Table S1).

To induce differentiation into adipocytes, DPSCs and ADSCs were cultured for 21 days with two different adipogenic media, described in the Materials and Methods Section. Although lipid vacuoles were observed after 14 and 21 days of culture for ADSCs in both differentiation media, no such vacuoles were observed in the DPSCs cultured under the same conditions (Figure 2A). After Oil Red O staining, DPSCs cultures appeared similar to the negative control sample, which did not receive differentiation induction media, with no stained lipid vacuoles observed in the samples subjected to adipogenic induction (Figure 2B). The same can be observed after quantification of cells stained with Oil red O after adipogenic differentiation using commercial culture medium (medium 1—M1) and custom culture medium (medium 2—M2) (Table 1).

However, conclusions based only on Oil Red O staining can be subjective, and the method is subject to artefacts resulting from dye accumulation due to a lack of washing or filtration before staining, making it difficult to interpret the data or even leading to erroneous interpretations about the results [7,8,10,22]. In accordance with our results, Struys et al. [23] showed adipogenic differentiation in only 30% of the samples, and moreover, lipid vacuoles were detected in few cells. Other studies based their conclusion about DPSCs adipogenic differentiation on the expression of one molecular marker, even though Oil Red O-stained lipid vacuoles were not found, and concluded that differentiation can occur later in DPSCs, after 5 weeks [24,25]. All these observations and our data indicate that DPSCs have limited or no capacity for adipogenesis. At the molecular level, FABP4, a commonly used adipogenic marker, was highly expressed in the positive control samples (induced ADSCs) in both induction media, commercial culture medium (M1) and custom culture medium (M2), with a clear correlation between FABP4 protein expression and lipid droplets, as demonstrated by Eom et al. [26]. One of the induced DPSCs samples showed expression of FABP4. However, expression of the PPARG gene, a master regulator of adipogenesis [27], was found only in the induced ADSCs samples (Supplementary Figure S2). Basing our interpretation on a single reverse transcription-polymerase chain reaction (RT-PCR) product could have led us to an erroneous conclusion about the adipogenic differentiation potential of DPSCs. FABP4 gene expression has been detected in cell types other than adipocytes and, more importantly, in murine lymphocytes and squamous cell carcinoma treated with dexamethasone, as a response to the chemical and not as part of an adipocyte differentiation process [28,29]. This response to dexamethasone could explain the expression of FABP4 without correlation with the presence of lipid vacuoles in the Oil Red O staining since dexamethasone is one of the common induction agents used in our work and in most, if not all, adipogenesis media. One other explanation might be related to the fact that our observation was at the mRNA level and does not necessarily reflect the FABP4 protein levels.

**Figure 3:**
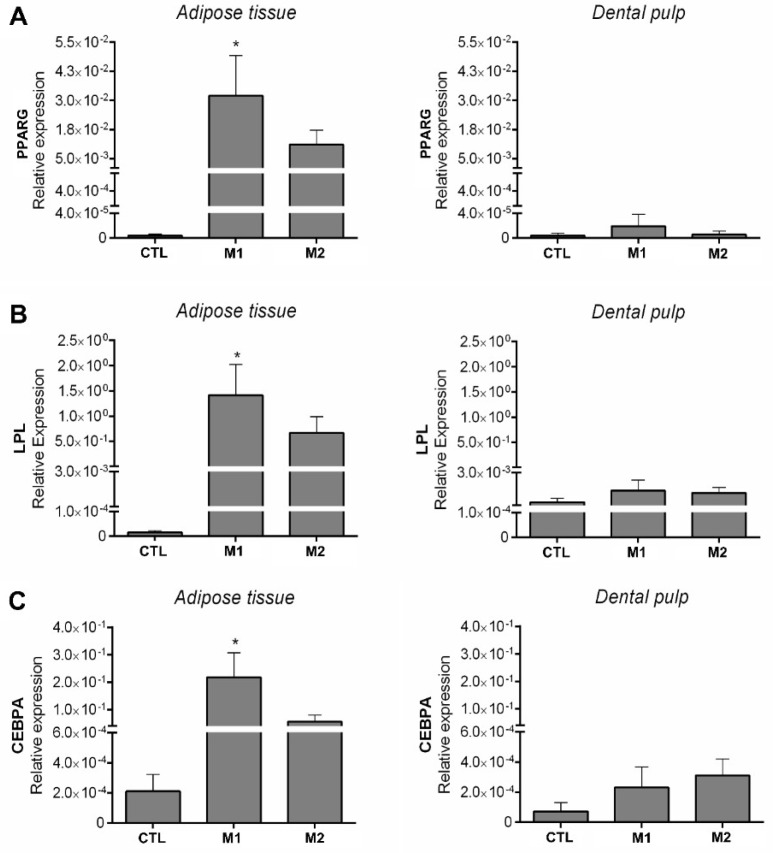
Expression of PPARG, LPL and CEBPA by qRT-PCR. The mRNA levels of markers of adipogenesis were measured by quantitative real-time PCR and normalised to GAPDH levels. (**A**) Expression of PPARG in ADSCs and DPSCs, (**B**) expression of LPL in ADSCs and DPSCs, (**C**) expression of CEBPA in ADSCs and DPSCs. CTL: control; M1: commercial culture medium; M2: custom culture medium; PPARG: peroxisome proliferator-activated receptor gamma; LPL: lipoprotein lipase; CEBPA: CCAAT/enhancer-binding protein alpha; GAPDH: glyceraldehyde-3-phosphate dehydrogenase, housekeeping gene; ADSCs: adipose tissue-derived stromal cells; DPSCs: dental pulp-derived stromal cells. Mean with SEM; One-way analysis of variance (ANOVA), with multiple comparisons: * *p* <  0.05.

Since we have detected expression of FABP4 in one of the DPSCs samples by RT-PCR, we wondered whether PPARG and other markers of adipogenesis could be detected using another technique in these samples. Thus, we analysed the expression levels of PPARG, LPL and CCAAT/enhancer-binding protein alpha (CEBPA) by real-time quantitative reverse transcription-polymerase chain reaction (qRT-PCR) [30] (Figure 3). All three markers were highly expressed at the mRNA level in ADSCs in both differentiation media tested when compared to DPSCs. In addition, the mRNA levels increased in the induced ADSCs culture in comparison to the uninduced controls; however, only the increase in commercial medium (M1) was statistically significant (Figure 3 and Supplementary Figure S3). In accordance with our microscopic observations of adipogenesis, overall, no DPSCs samples exhibited high expression or clear induction of PPARG, LPL or CEBPA (qRT-PCR; Figure 3 and Supplementary Figure S3). PPARG is an essential regulator of adipogenesis both in vitro and in vivo. It has been shown that without PPARG expression, precursor cells are unable to differentiate into mature adipocytes [30,31,32]. CEBPA and PPARG are involved in a single adipogenic differentiation program, in which PPARG plays a dominant role while CEBPA is important in the terminal differentiation of adipocytes [30]. LPL is a central enzyme in lipoprotein metabolism and is important to adipocyte development [33]. Several cell types have low LPL expression, with only adipocytes and muscle cells expressing high levels of LPL [33]. Overall, our data demonstrate that DPSCs have a reduced potential for adipogenic differentiation. Moreover, we suggest that concluding that DPSCs differentiate into adipocytes based only on Oil Red O staining [7,22,34], particularly when no expression of marker genes is detected (e.g., PPARG and LPL, [8]), or based only on the expression of FABP4, may be premature. We believe that a more rigorous analysis must be conducted to confirm that a stromal cell has differentiated into a specific cell lineage. In this case, microscopic observation, two different induction media, Oil Red O staining and at least two molecular markers were necessary to determine whether DPSCs have the potential to differentiate into adipocytes.

### 2.3. Transcriptomic Analyses Revealed Basal Differences between ADSCs and DPSCs and the Inability of DPSCs to Undergo Adipogenic Differentiation

**Figure 4:**
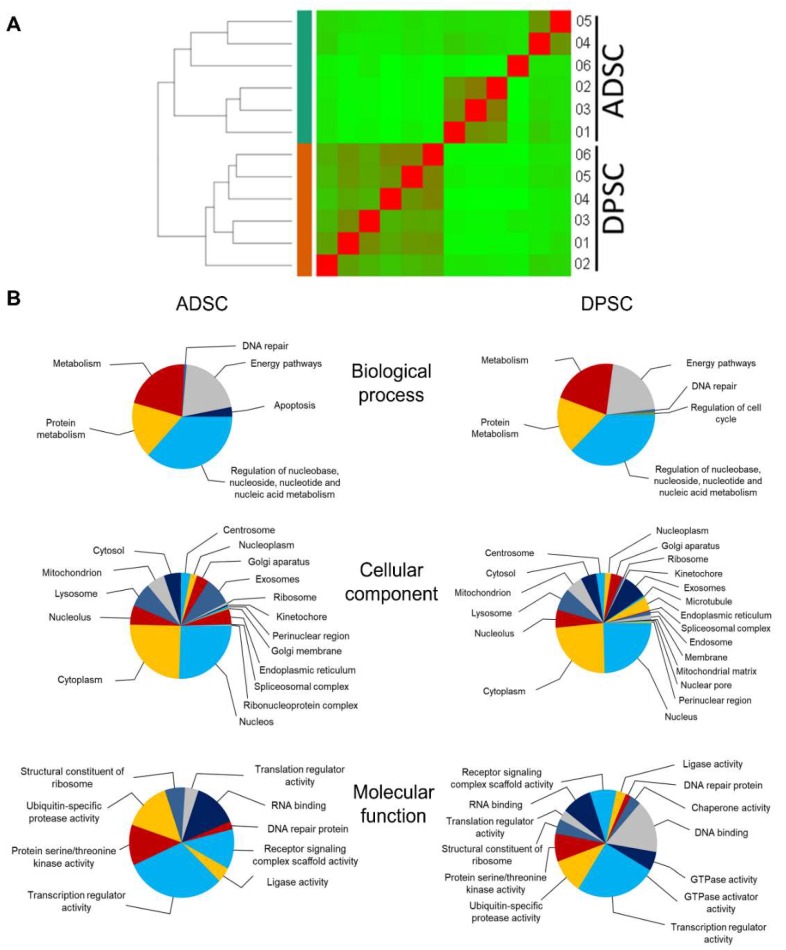
Hierarchical clustering and functional enrichment analyses of mRNAs present in ADSCs and DPSCs. (**A**) Hierarchical clustering based on RNA sequencing. (**B**) Gene Ontology (GO) enrichment analysis of all genes identified in ADSCs and DPSCs showing the most enriched terms for biological process, cellular component and molecular function. The pie chart shows selected significantly enriched categories (*p*-value < 0.05 for biological process and *p*-value < 0.01 for cellular components and molecular function). GO analysis was conducted with Funrich software. mRNA: messenger RNA; ADSCs: adipose tissue-derived stromal cells; DPSCs: dental pulp-derived stromal cells; RNA: ribonucleic acid; GO: Gene ontology.

To elucidate why DPSCs have no or low capacity for adipogenic differentiation, transcriptional profiles comparing DPSCs and ADSCs were performed by deep sequencing mRNA from six samples of each source. After mapping and filtering out mRNAs with low counts, 16,369 genes were retrieved per comparison. Hierarchical clustering shows that samples separate as a function of source (adipose tissue or dental pulp) rather than in a stochastic distribution (Figure 4A). Most transcripts, 14,871, were detected in both cell sources (though with different levels of expression), while fewer than 10% of the total number of transcripts detected were present in only one of the RNA populations, 1032 in ADSCs and 471 in DPSCs. This finding demonstrates that DPSCs and ADSCs are, for the most part, qualitatively similar. Accordingly, functional enrichment analyses conducted to determine the functions of those genes using Funrich software also showed similarity between the enriched Gene Ontology (GO) terms found for DPSCs and ADSCs. The significantly enriched biological process GO terms common to both samples were related to metabolism (nucleic acid, protein and energy pathways) and DNA repair (Figure 4B). One GO term in each source differentiated both cell types. The GO term significantly represented only in DPSCs was regulation of the cell cycle. Relatedly, it has been shown that DPSCs have a higher rate of proliferation than MSCs isolated from other sources, such as human bone marrow [6,8]. For ADSCs, the distinct GO term was apoptosis. Although the percentages of apoptotic cells were close to the lower detection level of the cytometer, the percentage of apoptotic cells was four times lower in DPSCs (0.33% ± 0.23%) than in ADSCs (1.46% ± 1.15%) (Figure 1).

**Figure 5:**
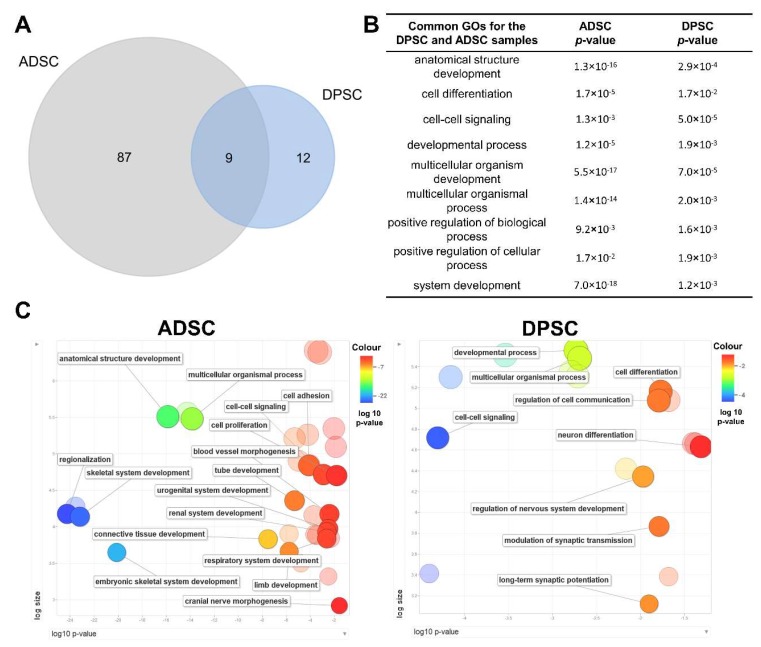
Functional enrichment analyses of DEGs in ADSCs and DPSCs. (**A**) Venn diagram of the GO terms for biological process terms identified in ADSCs and DPSCs samples. The diagram shows an overlap in GO terms that were common between the ADSCs and DPSCs. (**B**) GO terms that were common between the ADSCs and DPSCs with *p*-values. (**C**) GO enrichment analysis summarised and visualised as a scatter plot using REVIGO. Summarised exclusive set of GO terms related to biological process in the ADSCs and DPSCs. GO terms were ordered in relation to the *p*-value (x-axis) obtained from GO term enrichment analysis and the frequency of GO terms in the Gene Ontology Annotation Database (y-axis). Bubble colour indicates the provided *p*-value. DEGs: differentially expressed genes; ADSCs: adipose tissue-derived stromal cells; DPSCs: dental pulp-derived stromal cells; GO: Gene Ontology.

To obtain greater insight into the differences between cell types, a stringent and conservative analysis was performed using the differentially expressed genes (DEGs) with fold changes (log2) > 4 or < –4 to determine the GO term characteristics of each cell type. A total of 510 genes were differentially expressed: 141 were upregulated in DPSCs, and 369 were upregulated in ADSCs. The lists of GO terms for biological processes from each source with significant *p*-values were compared. Nine terms were common to both cell types and could be considered very characteristic of a multipotent stromal cell, as these terms were associated with the expression of genes involved in cell signalling and cell differentiation (Figure 5A,B). Because the number of GO terms obtained per category was quite high, the list of GO terms and their respective *p*-values for each cell type was summarised using REVIGO (Figure 5C).

ADSCs showed a variety of GO terms for biological processes related to different developmental and differentiation processes. Connective tissue development was a GO term highly significantly represented (*p* value = 2.98 × 10^−8^) in ADSCs and absent in the DPSCs GO list. Interestingly, neural and neural-related differentiation processes were overrepresented in DPSCs, and a diversity of other cell differentiation and developmental processes were overrepresented in ADSCs, e.g., urogenital system development, renal system development and respiratory system development. It is worth pointing out that the transcripts involved in developmental processes other than the neural system were also present in DPSCs but at much lower levels than in ADSCs. Together, these observations may indicate that DPSCs have more restricted potential than ADSCs and might reflect the shared ectodermal/neural crest origin of DPSCs and neural tissues.

Remarkably, several of the transcripts identified as differentially represented in each cell source are parts of signalling pathways already known to be responsible for inhibiting or stimulating adipogenic differentiation (Table 2).

The canonical WNT pathway responsible for regulating cell growth and commitment, when activated, inhibits adipogenic differentiation [12,13,35]. Some genes can block this pathway, favouring adipogenesis, such as Dickkopf WNT signalling pathway inhibitor 1 (DKK1) and secreted frizzled-related protein 4 (sFRP4). DKK1 is overrepresented in ADSCs, contributing to its favourable adipogenic differentiation. Another gene of the WNT pathway related to adipogenesis, transcription factor 4 (TCF4), was also upregulated in ADSCs—when expressed in preadipocytes, TCF4 promotes differentiation into adipocytes [36]. Genes still related to the WNT pathway but responsible for the inhibition of adipogenic differentiation were overrepresented in DPSCs. The wingless-type, member 10B (WNT10B) gene [13,37] prevents adipogenic differentiation by blocking the expression of key adipogenic transcription factors, such as PPARG and CEBPA [12]. In our transcriptome data, a significant increase in WNT10B expression was observed in DPSCs, at least partially explaining the origin of the limited capacity of DPSCs for differentiation and the possible absence of the PPARG transcript after 21 days of adipogenic induction (Figure 1).

The WNT pathway blocks adipogenesis and promotes proliferation [35]. We obtained results consistent with these activities and observed that genes expressed later in the WNT pathway are expressed in DPSCs, which continue to proliferate even in adipogenic differentiation conditions, indicating that this pathway related to cell growth is not blocked in DPSCs. Concordantly, we observed that when the same numbers of cells were plated from both sources (80% confluence), DPSCs achieved 100% confluence after 14 days of adipogenic induction culture, while ADSCs differentiated into adipocytes but did not proliferate, maintaining the original confluence.

Another pathway responsible for adipogenesis is the BMP pathway, which is part of the transforming growth factor beta (TGF-β) superfamily [27]. This pathway regulates MSCs differentiation in cartilage, bone tissue, tendon, ligament and adipogenic lineages [35]. Noggin (NOG), a BMP antagonist, acts by blocking this pathway [16], and in this study, NOG was expressed at significantly higher levels in DPSCs than in ADSCs. Other genes in the BMP pathway, when upregulated, play important roles in adipogenesis, such as the inhibitors of DNA-binding (ID)-1, -2 and -3 genes. These IDs are found at higher levels in ADSCs, thus contributing to adipogenesis [27].

Independent of these two signalling pathways, the Msh-like 2 homeobox (MSX2) gene is an important transcriptional regulator for the commitment of mesenchymal stromal cells into osteoblasts and adipocytes. MSX2 promotes osteoblast differentiation independently of runt-related transcription factor 2 (RUNX2) and negatively regulates adipocyte differentiation through inhibition of PPARG and the C/EBP family, resulting in inhibition of adipogenesis [27,38]. This gene is overrepresented in DPSCs, possibly suppressing the adipocyte differentiation of mesenchymal cells.

## 3. Materials and Methods

### 3.1. Isolation and Cell Cultivation

This project was approved by the research ethics committee of the Pontifical Catholic University of Parana (CAAE: 42751615.8.0000.0020, date of approval: 4 May 2015). All samples were collected after obtaining a completed informed consent form.

Samples of dental pulp from the third molar (*n* = 6) and samples of adipose tissue from liposuction (*n* = 6) were used.

To collect permanent teeth, the dentist previously requested that the patient use a mouth rinse containing chlorhexidine to remove possible contaminants, ensuring the integrity of the collected material. The teeth were washed in phosphate-buffered saline solution (PBS) in a petri dish, and the tooth pulp fragments were mechanically removed with a K file and macerated. The material was dissociated by the action of collagenase type II (1 mg/mL—Invitrogen, Auckland, New Zealand) under stirring at 37 °C for 1 h and subsequently filtered and centrifuged in PBS. The cells were resuspended and plated in flasks with Iscove’s Modified Dulbecco’s Media (IMDM) (Invitrogen, Auckland, New Zealand) supplemented with 1% antibiotic (penicillin/streptomycin—Invitrogen, Auckland, New Zealand) and 15% foetal bovine serum (FBS) (Invitrogen, Auckland, New Zealand). Cultures were stored in an incubator at 37 °C with 5% CO_2_.

Adipose tissue was obtained from donors who underwent liposuction. ADSCs were isolated using the enzymatic digestion method. Briefly, 100 mL of adipose tissue was washed with PBS, and the tissue was digested with collagenase type I (Invitrogen, Auckland, New Zealand) for 30 minutes at 37 °C under stirring, followed by filtration using 100 and 40 μm filters. Cells were cultured in DMEM-F12 Dulbecco’s Modified Eagle Medium: Nutrient Mixture F-12 (DMEM-F12) (Invitrogen, Auckland, New Zealand) supplemented with 1% antibiotic (penicillin/streptomycin—Invitrogen, Auckland, New Zealand) and 15% FBS (Invitrogen, Auckland, New Zealand). Cultures were stored in an incubator at 37 °C with 5% CO_2_.

The culture medium was replaced twice a week. When the cultures reached approximately 80–90% confluence, cells were dissociated using 0.25% trypsin/EDTA (Invitrogen, Auckland, New Zealand) and re-plated (passage 1).

### 3.2. Immunophenotypic Characterisation

For the immunophenotypic characterisation of DPSCs (*n* = 3, passage four) and ADSCs (*n* = 3, passage four), commercial antibodies were used to analyse the expression of cell surface markers. The labelling was performed according to Rebelatto et al. [21]. Briefly, the cells were washed with PBS and incubated in the dark for 30 minutes with CD29-APC (1:20), CD14-FITC (1:20), CD45-FITC (1:20), CD19-FITC (1:20), CD34-APC (1:20), CD56-PE (1:10), CD105-APC (1:20), CD73-APC (1:33), CD90-PE (1:100), CD166-PerCP (1:33), annexin V (1:20) and 7-aminoactinomycin D (7-AAD)(1:20) (all markers and dyes used—Becton Dickinson, San Diego, CA, USA). The cells were washed with wash buffer and resuspended in a solution containing 1% formaldehyde. Isotypic IgG1 mouse antibodies were used as controls. Approximately 100,000 labelled cells were acquired by a FACSVerse flow cytometer (Becton Dickinson, Franklin Lakes, NJ, USA) and were analysed using FlowJo software with the default parameters (FlowJo, Ashland, OR, USA—version 8.1).

### 3.3. Osteogenic Differentiation

DPSCs (*n* = 3, passage (P) four) and ADSCs (*n* = 1, P4) were plated at a concentration of 20,000 cells/cm^2^ in triplicate in 24-well plates on glass coverslips. The cells were kept in an incubator at 37 °C with 5% CO_2_ until reaching 80% confluence. Commercial medium was used for osteogenic differentiation (Differentiation Basal Medium-Osteogenic, Lonza, Walkersville, MD, USA). For control, cells were cultured with IMDM supplemented with 15% FBS without differentiation factors or inducers. The cultures were maintained for three weeks, and the culture medium was replaced every two to three days. To evaluate the presence of calcium crystals, after fixation with 1% formaldehyde, the samples were stained with Alizarin Red at pH 4.1 (Sigma-Aldrich, St. Louis, MO, USA). Briefly, the cells were washed with PBS, fixed with 1% formaldehyde and stained with Alizarin Red.

### 3.4. Chondrogenic Differentiation

Micromass culture was performed for chondrogenic differentiation (ADSC, *n* = 1; DPSC, *n* = 3, both P4). Approximately 1 × 10^6^ cells in 1 mL of culture medium were centrifuged at 300× *g* for 10 min in a conical tube to form a cell pellet. The micromass was cultured with chondrogenic differentiation medium (Differentiation Basal Medium-Chondrogenic, Lonza, Walkersville, MD, USA) supplemented with TGF-β3 (Lonza, Walkersville, MD, USA). For control, cells were cultured with IMDM supplemented with 15% FBS without differentiation factors or inducers. The cultures were maintained for three weeks, and the culture medium was replaced every two to three days. On day 21, cell aggregates were fixed in 10% formaldehyde for 1 h at room temperature, dehydrated in serial ethanol dilutions, and embedded in paraffin blocks. Paraffin sections (4 µm thick) were stained for histologic analysis with Toluidine blue solution (Sigma-Aldrich, St. Louis, MO, USA) to evaluate the presence of intracellular matrix mucopolysaccharides.

### 3.5. Adipogenic Cell Differentiation

To evaluate the adipogenic potential, three samples of DPSCs and one of ADSCs (all at P4) were differentiated. DPSCs and ADSCs were plated at a concentration of 20,000 cells/cm^2^ in triplicate in 24-well plates on glass coverslips for cytochemistry.

For RT-PCR and qRT-PCR, the cells were plated in 6-well plates at the same concentration. Cells were kept in an incubator at 37 °C with 5% CO_2_ until reaching 80% confluence. Two culture media were used for differentiation: Medium 1 (M1): commercial culture medium (hMSC Adipogenic Differentiation Bullet Kit, Lonza, Walkersville, MD, USA); Medium 2 (M2): custom culture medium, DMEM supplemented with 10% FBS, 1 μM dexamethasone, 100 μM indomethacin, 1 μg/mL insulin and 500 μM 3-isobutyl-l-methylxanthine [39]. For the control, cells were cultured with IMDM supplemented with 15% FBS without differentiation factors or inducers. The cultures were maintained for three weeks, and the culture medium was replaced every two to three days. After adipogenic differentiation, Oil Red O dye (Sigma-Aldrich, St. Louis, MO, USA) was used to evaluate the presence of lipid vacuoles inside the cells. Briefly, the cells were fixed with 70% ethanol, washed with water, and stained with a 0.5% solution of Oil Red O. For nucleus staining, hematoxylin was used. Additionally, an Oil-Red quantification was performed. Twenty fields of view were randomly picked in two different wells (*n* = 40). The total number of cells and the number of Oil Red-positive cells were assessed, and numbers of Oil Red-positive cells were calculated as a percentage of total cells. 

### 3.6. Total RNA ExStraction and Reverse Transcription-Polymerase Chain Reaction (RT-PCR)

Total RNA was extracted with TRIzol Reagent (Invitrogen, Carlsbad, CA, USA) according to the manufacturer’s instructions. To degrade any contaminating DNA, RNA was treated with DNase I (Qiagen, Germantown, MD, USA). Complementary DNA (cDNA) was synthesised from 1 µg of total RNA using oligo-dT primers (USB Corporation, Cleveland, OH, USA) and a reverse transcriptase kit (ImPROm-II, Promega, Madison, WI, USA) according to the manufacturer’s instructions. Polymerase chain reaction (PCR) was carried out with 100 ng of cDNA as the template, 5 pmol of each primer, *Taq* polymerase and reaction mix (IBMP, Brazil). The following primers were used: PPARG (5′ATTACAGCAAACCCCTATTCC3′ and 5′GGCATCTCTGTGTCAACCAT3′) and FABP4 (5′ATGGGATGGAAAATCAACCA3′ and 5′GTGGAAGTGACGCCTTTCAT3′). We subjected 20 µL of the RT-PCR products to electrophoresis in a 2% agarose gel. The bands obtained were visualised by GelRed^®^ Nucleic Acid Gel Stain (Biotium, Fremont, CA, USA) and photographed under ultraviolet transillumination (UV White Darkroom, UVP Bioimaging Systems, Upland, CA, USA). Glyceraldehyde-3-phosphate dehydrogenase (GAPDH) transcript was used as an internal control (5′GGCGATGCTGGCGCTGAGTAC3′ and 5′TGGTTCACACCCATGACGA3′).

### 3.7. Total RNA Extraction and Quantitative Real-Time Polymerase Chain Reaction (qRT-PCR)

The mRNA levels of adipogenic marker genes, PPARG, CEBPA and LPL, were detected using qRT-PCR. Each qRT-PCR reaction was composed of sample cDNA (obtained as described in the previous section), 5 pmol of primer and 5 µL of GoTaq^®^ DNA Polymerase (Promega, Madison, WI, USA). GAPDH was used as an endogenous control. The qRT-PCR conditions were as follows: 95 °C for 120 s, 45 cycles of 95 °C for 15 s, 60 °C for 15 s and 60 °C for 30 s, followed by 95 °C for 10 s, 65 °C for 60 s and 95 °C for 15 s. PCRs without template were used as the negative control. For comparative analysis of gene expression, the relative expression levels were obtained by either the normalisation with GAPDH mRNA level or the threshold PCR cycle (Ct) determined using the 2^−ΔΔCt^ analysis method. Each gene was analysed in three technical replicates for each biological sample. The primer sequences for PPARG were forward: 5′ATTACAGCAAACCCCTATTCC3′ and reverse: 5′GGCATCTCTGTGTCAACCAT3′. The QuantiTect Primer Assays Hs_GAPDH_1_SG, Hs_CEBPA_1_SG and Hs_LPL_1_SG were acquired from Qiagen (USA).

### 3.8. RNA Sequencing

To compare the phenotype of the undifferentiated cells, RNA extraction was performed from DPSCs (*n* = 6) and ADSCs (*n* = 6), between passages three and six, according to the protocol described in a commercial kit (PureLink RNA Mini Kit—Life Technologies, Carlsbad, CA, USA). Cell lysis, purification and RNA elution were performed.

Samples were treated with DNase (RNase-Free DNase Set- Qiagen, Germantown, MD, USA). The RNA was quantified in a spectrophotometer (Nanodrop-Thermo Scientific, Wilmington, DE, USA), and the quality was analysed with Bioanalyzer equipment (Agilent, Santa Clara, CA, USA). Only samples with an RNA integrity number (RIN) better than 7 were considered for sequencing.

Samples were sent to the Edinburgh Genomics sequencing facility (Edinburgh, UK) and multiplexed and sequenced on Illumina HiSeq 4000 equipment (Illumina Inc., San Diego, CA, USA) to generate paired-end fragments of 100 base pairs.

### 3.9. Data Availability

The raw data from all RNA-Sequencing (RNA-Seq) samples used to support the findings of this study have been deposited in an NCBI-BioProject (project code PRJNA516691), which provides users a single place to find a link to the diverse data types generated for the project.

### 3.10. Bioinformatics Analysis of RNA-Seq Data

Raw sequenced transcriptome libraries (RNA-Seq) were filtered for high-quality read selection based on average read quality and per position nucleotide detection using NGS QC Toolkit software [40]. High-quality reads were mapped to the human reference genome (GRCh38) using Star aligner [41], producing compressed binary BAM files. Next, the binary BAM files were used in the HTSeq software package to account for the absolute number of mapped reads per annotated transcript (Ensembl GRCh38 annotation) in GRCh38, generating a count data matrix. The matrix was normalised by a read counting approach followed by a negative binomial distribution and Fischer’s exact statistical test for differential gene expression (DGE) analysis using the R Bioconductor package DESeq2 [42]. The FDR based on the Benjamini and Hochberg [43] method was used for the DGE genes to calculate the statistical significance between samples, with DGE transcripts having a *p*-value < 0.05.

The count data matrix for all sequenced samples was also used to calculate and generate a Euclidian distance matrix for hierarchical sample clustering to group samples according to the most similar transcriptome profile, including biological replicates, for sample clustering analysis. This method was used to generate a heatmap representing how sequenced samples are correlated. The normalised count matrix was also used for principal component analysis (PCA) using the R library ggplots from the Deseq2/Bioconductor package. Additionally, the single linkage method was used to generate a dendrogram and a heatmap showing the correlation of all sample expression profiles as colours ranging from green (most different) to red (identical profiles). Quantitative differences in differentially expressed transcripts between different developmental stages are represented in Venn diagrams to identify common or exclusive genes among stages.

### 3.11. Gene Ontology Annotation

Functional enrichment analysis was performed to obtain the biological processes, molecular functions and cellular components in which the identified proteins are involved. The Funrich (version 3.1.3) [44], G:profiler (University of Tartu, Tartu, Estonia—2005–2018 version) [45] and REVIGO (Rudjer Boskovic Institute, Zagreb, Croatia - 2017 version) [46]. Software/web servers were used with default settings.

## 4. Conclusions

If we strictly follow the guidelines outlined in the ISCT’s position paper on the minimum criteria for defining multipotent MSCs [1], our data indicated that DPSCs would poorly meet the MSC definition criteria. DPSCs do not have potential or have diminished potential for adipogenic differentiation, a requirement to be considered an MSCs. Correspondingly, overall, the gene expression profile showed relative overexpressions of a number of pro-adipogenic factors in ADSCs and anti-adipogenic factors in DPSCs, supporting our in vitro differentiation observations. In addition, the mRNA levels of genes involved in several other differentiation or development pathways were significantly higher in ADSCs than in DPSCs. However, we believe that even with the apparent reduced multipotentiality of DPSCs compared with ADSCs, it is important to further study DPSCs and to keep them in public/private cell banks. DPSCs have a high proliferative capacity and a likely high potential for differentiation into neural lines and/or for paracrine activity favouring neurogenesis for two reasons: DPSCs are ectodermal in origin, and the only group of overexpressed transcripts related to differentiation in DPSCs is associated with neural synapses and neural differentiation.

## Figures and Tables

**Table 1:** Descriptive percentage values of Oil Red O quantification.

Cell Population	Passage	Cell Culture Media	*n*	Mean (%)	Median (%)	SD (%)	Minimum Value (%)	Maximum Value (%)
DPSCs	P4	M1	40	0	0	0	0	0
		M2	40	0	0	0	0	0
ADSCs	P4	M1	40	49.2	50	18.3	20	100
		M2	40	48.5	47.4	11.9	24	78.3

ADSCs: adipose tissue-derived stromal cells, DPSCs: dental pulp-derived stromal cells, P: passage M1: medium 1, commercial culture medium, M2: medium 2, custom culture medium, *n*: number of quantified fields, SD: standard deviation.

**Table 2:** DEGs involved in adipogenesis.

	Gene	Expression in ADSCs	Expression in DPSCs	log2-fold-change	*p*-Value	padj.
**WNT Pathway**	*JUN*	12,495.79	28,397.18	1.14	0.020	0.08
	*CCND1*	30,864.96	106,032.86	1.70	0.001	7.5 × 10^−3^
	*DKK1*	8539.62	2376.18	−1.68	0.019	0.08
	*TCF4*	9071.10	2716.28	−1.68	2.09 × 10^−4^	0.002
	*MMP7*	0.52	6.15	2.63	0.015	0.07
	*WNT10B*	15.34	44.36	1.42	0.028	0.10
**BMP Pathway**	*ID1*	13,706.57	4008.96	−1.68	0.002	0.02
	*ID2*	21,168.96	2332.81	−2.98	1.11 × 10^−6^	2.67 × 10^−5^
	*ID3*	36,749.25	4789.64	−2.82	1.35 × 10^−8^	5.42 × 10^−7^
	*MSX2*	348.26	4816.06	3.40	6.04 × 10^−6^	1.2 × 10^−4^
	*NOG*	101.84	530.68	2.31	9.24 × 10^−8^	2.94 × 10^−6^

Genes of the WNT and BMP pathways had significant gene expression alterations (*p*-value < 0.05) in stem cells from adipose tissue (ADSCs) compared to those from dental pulp (DPSCs). Gene expression is shown as normalised count-based values. Log2 (fold-change) corresponds to the Log2 scale of the calculated fold-change between the expression found in DPSCs and the expression found in ADSCs. BMP: bone morphogenetic protein; ADSCs: adipose tissue-derived stromal cells; DPSCs: dental pulp-derived stromal cells; padj.: statistical *p*-value significance with false discovery rate (FDR) correction; DEGs: differentially expressed genes.

## Supplementary Materials

Supplementary materials can be found at https://www.mdpi.com/1422-0067/21/8/2753/s1.

## Abbreviations

MSCs	Mesenchymal Stromal Cells
ADSCs	Adipose Tissue-Derived Stromal Cells
DPSCs	Dental Pulp-Derived Stromal Cells
PPARG	Peroxisome Proliferator-Activated Receptor Gamma
LPL	Lipoprotein Lipase
BMP	Bone Morphogenetic Protein
ISCT	International Society for Cellular Therapy
FABP4	Fatty Acid Binding Protein 4
M1	Commercial Culture Medium
M2	Custom Culture Medium
CEBPA	CCAAT/Enhancer-Binding Protein Alpha
PC	Positive Control
CTL	Control
GAPDH	Glyceraldehyde-3-phosphate Dehydrogenase
SEM	Standard Error of the Mean
P	Passage
RNA	Ribonucleic Acid
mRNA	Messenger RNA
DNA	Deoxyribonucleic Acid
GO	Gene Ontology
DEGs	Differentially Expressed Genes
FDR	False Discovery Rate
DKK1	Dickkopf WNT Signalling Pathway Inhibitor 1
sFRP4	Frizzled-Related Protein 4
TCF4	Transcription Factor 4
WNT10B	Wingless-Type, Member 10B
TGF-β	Transforming Growth Factor Beta
NOG	Noggin
ID	DNA-binding
MSX2	Msh-Like 2 Homeobox
RUNX2	Runt-Related Transcription Factor 2
PBS	Phosphate-Buffered Saline Solution
IMDM	Iscove’s Modified Dulbecco’s Media
FBS	Foetal Bovine Serum
DMEM-F12	Dulbecco’s Modified Eagle Medium: Nutrient Mixture F-12
7-AAD	7-Aminoactinomycin D
hMSC	Human Mesenchymal Stromal Cells
RT-PCR	Reverse Transcription-Polymerase Chain Reaction
cDNA	Complementary DNA
PCR	Polymerase Chain Reaction
qRT-PCR	Quantitative Real-Time Polymerase Chain Reaction
