# Supplementary material for: The Expression Profile of Dental Pulp-Derived Stromal Cells Supports Their Limited Capacity to Differentiate into Adipogenic Cells
Source: Int J Mol Sci. 2020 Apr 15;21(8):2753. doi: 10.3390/ijms21082753 (PMC7215853; doi:10.3390/ijms21082753)
Supplement: Supplementary file 1 [file ijms-21-02753-s001.pdf]

Supplementary Materials

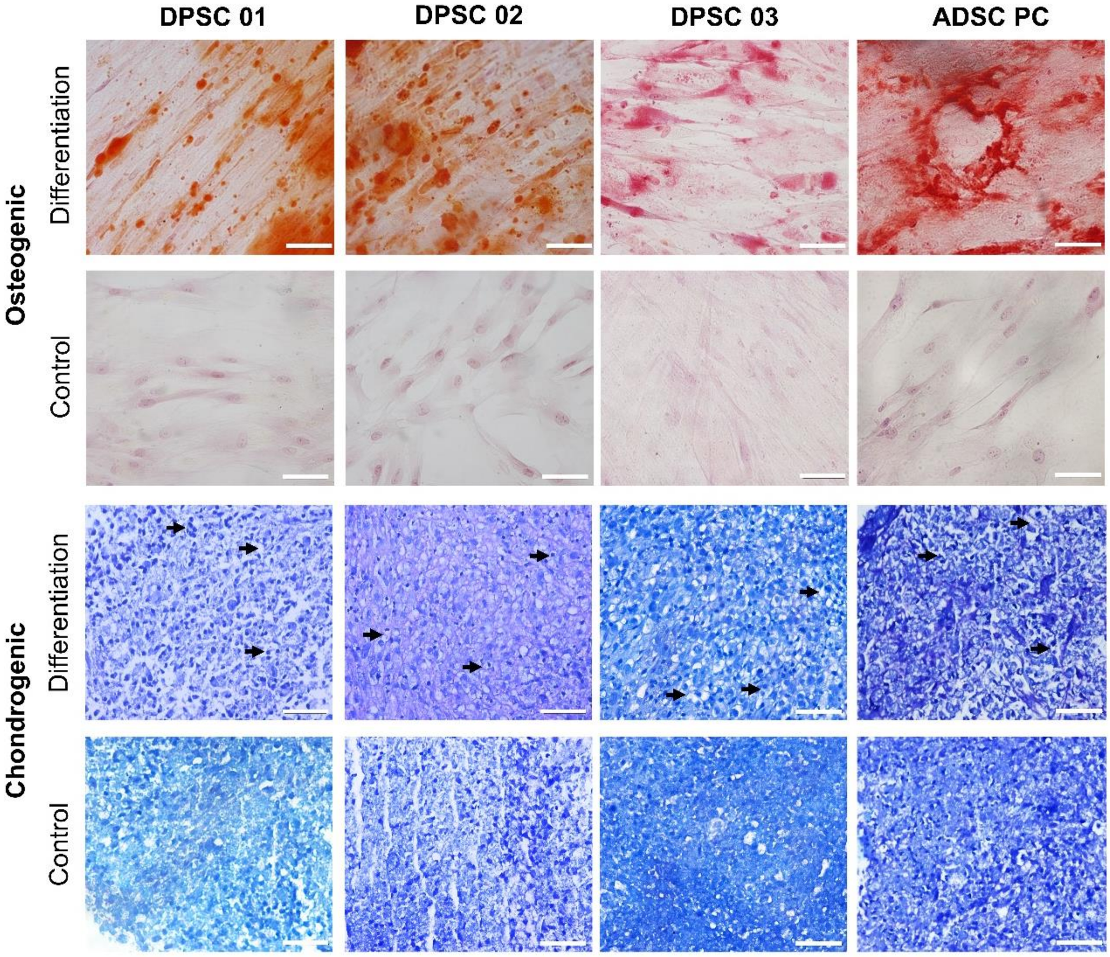

Figure S1. In vitro osteogenic and chondrogenic differentiation. Comparison between the positive control (ADSC) and three samples of DPSCs. Osteogenic differentiation: characterised by the presence of calcium deposits when stained with Alizarin Red S. Chondrogenic differentiation: characterized by the presence of vacuoles around young chondrocytes (arrows) staining with toluidine blue at the cartilaginous matrix. Scale bar: 50 μm. ADSC: adipose tissue-derived stromal cells; DPSC: dental pulp-derived stromal cells.

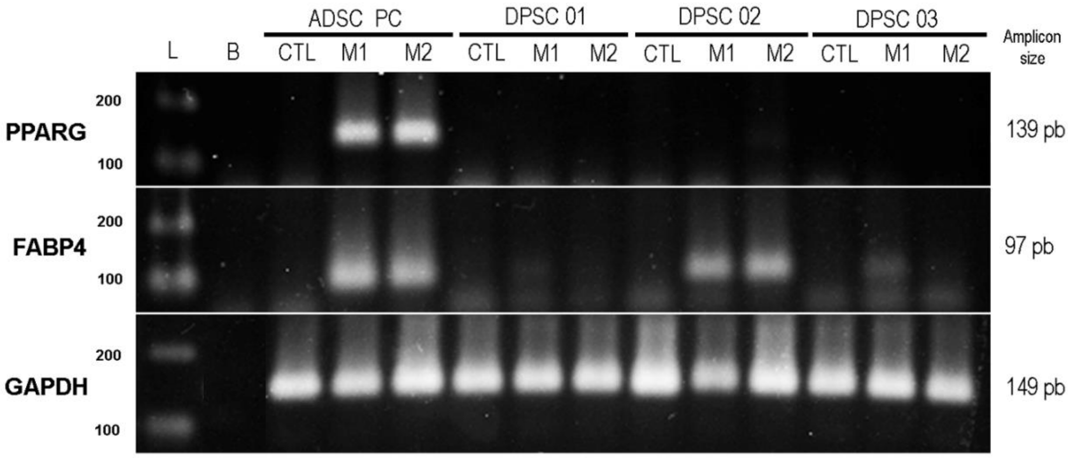

Figure S2. Expression of the PPARG and FABP4 markers of adipogenesis by RT-PCR. Comparison between the positive control (ADSCs) and three samples of DPSCs. CTL: control; M1: commercial culture medium; M2: custom culture medium; L: ladder, B: blank; PPARG: peroxisome proliferator-

activated receptor gamma; FABP4: fatty acid binding protein 4; GAPDH: glyceraldehyde-3-phosphate dehydrogenase, housekeeping gene; ADSC: adipose tissue-derived stromal cells; DPSC: dental pulp-derived stromal cells.

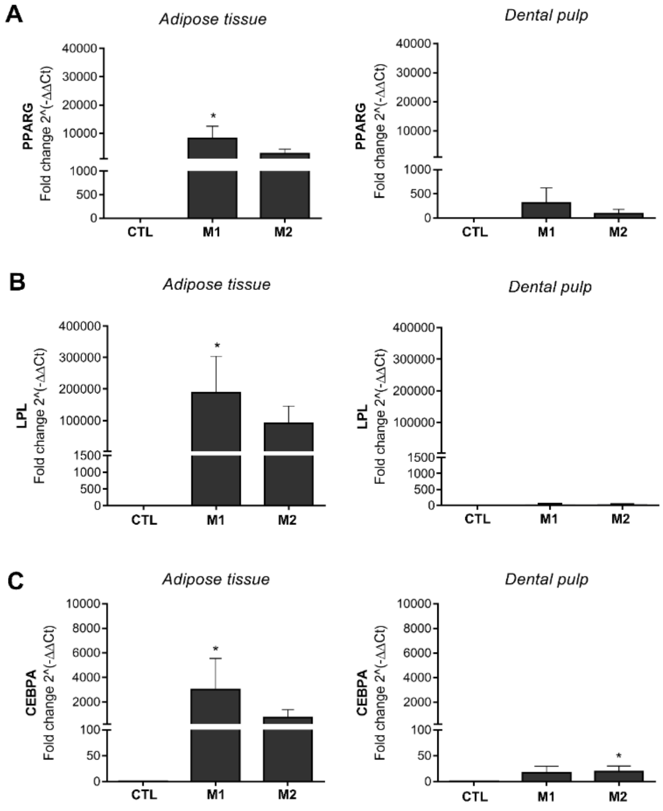

Figure S3. Expression of markers of adipogenesis by qRT-PCR. Fold changes (q-PCR $2^{-\Delta\Delta Ct}$ method) of (**A**) PPARG, (**B**) LPL and (**C**) CEBPA in ADSCs and DPSCs. CTL: control; M1: commercial culture medium; M2: custom culture medium; PPARG: peroxisome proliferator-activated receptor gamma; LPL: lipoprotein lipase; CEBPA: CCAAT/enhancer-binding protein alpha; ADSCs: adipose tissue-derived stromal cells; DPSCs: dental pulp-derived stromal cells. Values are given as fold change ± SEM; One-way ANOVA, with multiple comparisons: *$p < 0.05$.

Table S1. Published data on DPSC adipogenesis potential.

Authors	Isolation: method	Differentiation according to authors	Induction medium	Induction time	Evaluation method	
					Staining	Other technique
Gronthos et al., 2000 [6]	3 mg/mL Collagenase type I and 4 mg/mL dispase for 1 hour at 37 °C	No	*L-ascorbate-2-phosphate;<br>*dexamethasone with inorganic phosphate	6 weeks	No	No
Gronthos et al., 2002 [24]	3 mg/mL Collagenase type I and 4 mg/mL dispase for 1 hour at 37 °C	Yes	* 0.5 mM isobutylmethylxanthine<br>* 0.5 μM hydrocortisone<br>* 60 μM indomethacin	5 weeks	Oil red	RT-PCR: PPARG2 and LPL (control: GAPDH)
Struys et al., 2011 [23]	Explants. The explants were left undisturbed for 14 days to allow the migration of cells.	Oil red staining did not reveal any intracellular lipid droplets following the adipogenic differentiation of DPSC, with the exception of 30% of the tests, in which a few Oil red positive cells were observed	Adipogenic differentiation medium (R&D Systems)	3 weeks	Oil red	Immunocytochemistry (FABP)
Tamaki et al., 2013 [7]	3 mg/mL collagenase type I and 4 mg/mL dispase for 1 hour at 37 °C	Yes	α MEM containing 10% FBS<br>* 0.5 mM isobutylmethylxanthine 1<br>* 0.5 μM hydrocortisone<br>* 60 μM indomethacin	3 weeks	Oil red	–
Chang et al., 2014 [47]	3 mg/mL Collagenase type I and 4 mg/mL dispase for 1 hour at 37 °C	Yes	–	15 days	Oil red	PCR: PPARG and LPL
Kumar et al., 2015 [22]	Explant	Yes	αMEM supplemented with 10 % FBS<br>* 0.5 mM isobutyl-methylxanthine (IBMX)<br>* 1 μM dexamethasone<br>* 200 μM indomethacin<br>* 10 μM insulin	3 weeks	Oil red	Oil red O quantification by ELISA
Alsulaimani et al., 2016 [9]	Collagenase type I (250 units/mg) freshly mixed with 1 mL of dispase (5000 caseinolytic units in 100 mL)	Yes	DMEM supplemented with 10% FBS<br>* 10% horse serum<br>* 1% pen-strep<br>* 100 nM dexamethasone<br>* 0.45 mM isobutyl methylxanthine<br>* 3 μg/mL insulin<br>* 1 μM rosiglitazone	7, 14 and 21 days	Oil red	RT-PCR: PPAR-G2 and activated protein-2

Isobe et al., 2016 [8]	0.3% Collagenase type I and 0.4% dispase 1 hour at 37 °C	Yes	Induction medium<br>*αMEM com 20% FBS<br>*2 mM L-glutamine<br>*60 μM indomethacin<br>*100 μM L-ascorbic acid 2-phosphate<br>*0.5 mM isobutyl methylxanthine<br>*0.5 μM hydrocortisone<br>*10 μg/mL insulin<br>*100 U/ml pen/strep<br>Maintenance medium<br>*αMEM supplemented with 20% FBS<br>*2 mM L-glutamine<br>*100 μM L-ascorbic acid 2-phosphate<br>*10 μg/mL insulin *100 U/mL pen/strep	28 days	Oil red	RT-PCR: PPARG2 and LPL (control: GAPDH)
Ullah et al., 2016 [10]	PBS supplemented with 1 mg/mL collagenase type I at 37 °C with frequent shaking for 40 minutes.	Yes	*1 μM dexamethasone<br>*10 μM insulin<br>*100 μM indomethacin<br>*500 μM isobutylmethyl xanthine (IBMX)	21 days	Oil red	RT-qPCR: FABP4, LPL and PPARG (Control: YWHAZ)
Yang et al., 2017 [34]	3 mg/mL collagenase type I and 4 mg/mL dispase for 1 hour at 37 °C	Yes	*10% FBS<br>*0.4 μg mL^{-1} dexamethasone<br>*5 μg mL^{-1} insulin<br>*111 μg mL^{-1}<br>*isobutylmethylxanthine<br>*72 μg mL^{-1} indomethacin	14 days	Oil red	–

References

47 Chang, C.-C.; Chang, K.-C.; Tsai, S.-J.; Chang, H.-H.; Lin, C.-P. Neurogenic differentiation of dental pulp stem cells to neuron-like cells in dopaminergic and motor neuronal inductive media. *Journal of the Formosan Medical Association* **2014**, *113*, 956-965, doi:https://doi.org/10.1016/j.jfma.2014.09.003.
